# Zoonotic Viruses Associated with Illegally Imported Wildlife Products

**DOI:** 10.1371/journal.pone.0029505

**Published:** 2012-01-10

**Authors:** Kristine M. Smith, Simon J. Anthony, William M. Switzer, Jonathan H. Epstein, Tracie Seimon, Hongwei Jia, Maria D. Sanchez, Thanh Thao Huynh, G. Gale Galland, Sheryl E. Shapiro, Jonathan M. Sleeman, Denise McAloose, Margot Stuchin, George Amato, Sergios-Orestis Kolokotronis, W. Ian Lipkin, William B. Karesh, Peter Daszak, Nina Marano

**Affiliations:** 1 Wildlife Conservation Society, Bronx, New York, United States of America; 2 EcoHealth Alliance, New York, New York, United States of America; 3 Centers for Disease Control and Prevention, Atlanta, Georgia, United States of America; 4 Columbia University, New York, New York, United States of America; 5 Cummings School of Veterinary Medicine, Tufts University, North Grafton, Massachusetts, United States of America; 6 United States Geological Survey National Wildlife Health Center, Madison, Wisconsin, United States of America; 7 Sackler Institute for Comparative Genomics, American Museum of Natural History, New York, New York, United States of America; Global Viral Forecasting Initiative, United States of America

## Abstract

The global trade in wildlife has historically contributed to the emergence and spread of infectious diseases. The United States is the world's largest importer of wildlife and wildlife products, yet minimal pathogen surveillance has precluded assessment of the health risks posed by this practice. This report details the findings of a pilot project to establish surveillance methodology for zoonotic agents in confiscated wildlife products. Initial findings from samples collected at several international airports identified parts originating from nonhuman primate (NHP) and rodent species, including baboon, chimpanzee, mangabey, guenon, green monkey, cane rat and rat. Pathogen screening identified retroviruses (simian foamy virus) and/or herpesviruses (cytomegalovirus and lymphocryptovirus) in the NHP samples. These results are the first demonstration that illegal bushmeat importation into the United States could act as a conduit for pathogen spread, and suggest that implementation of disease surveillance of the wildlife trade will help facilitate prevention of disease emergence.

## Introduction

No adequate estimate of numbers of wildlife traded throughout the world exists given the large size and covert nature of the business. Beyond the threats to conservation, the intermingling of wildlife, domestic animals and humans during the process of wildlife extraction, consumption, and trade can serve as a vessel for pathogen exchange [1]. Nearly 75% of emerging infectious diseases in humans are of zoonotic origin, the majority of which originate in wildlife [2], [3]. Therefore infectious diseases acquired from contact with wildlife, such as occurs via the wildlife trade, are increasingly of concern to global public health.

Trade in live animals and animal products has led to the emergence of several zoonotic pathogens, of which RNA viruses are the most common. SARS emerged as a respiratory and gastrointestinal disease in southwest China and within months had spread to 29 other countries, eventually leading to 8,098 cases and 774 deaths. Masked palm civets (*Paguma larvata*) traded in the markets of Guangdong were found to be infected and a large proportion of the early cases were restaurant workers who bought and butchered wildlife from these markets [4].

The United States is one of the world's largest consumers of imported wildlife and wildlife products [5]. Between 2000 and 2006, approximately 1.5 billion live wild animals (around 120,000,000 per year) were legally imported into the United States nearly 90% of which were destined for the pet industry [6], and an average of over 25 million kilograms of non-live wildlife enter the United States each year [5]. New York is the most frequently used port of entry into the United States, and in combination with Los Angeles and Miami accounts for more than half of all known wildlife imports. Imports most often refused entry (i.e., deemed to be illegal) into the United States included those from China, Philippines, Hong Kong, Thailand, and Nigeria [5] – countries with endemic pathogens such as highly pathogenic H5N1 influenza virus, Nipah virus, and simian retroviruses.

Health risks to the US public, agricultural industry, and native wildlife posed by the wildlife trade have generally not been quantified due to minimal surveillance of live animal imports and the absence of surveillance of wildlife product imports. Despite this, known examples of disease introductions to the United States via the wildlife trade have included pathogens of risk to wildlife, livestock and public health such as amphibian chytridiomycosis, exotic Newcastle's disease, and monkeypox, respectively. The monkeypox outbreak showed that a single shipment of infected animals can result in serious impact on public health, highlighting the challenges faced by agencies attempting to regulate both legal and illegal wildlife trade. The USDA regulates certain exotic ruminant species, some birds, some fish, a few species of tortoise, hedgehogs, tenrecs, and brushtail possums for specific foreign animal diseases to protect agricultural health. In general, there is no current remit for USDA to regulate species as potential threats to wildlife or public health. Species restricted by Centers for Disease Control and Prevention (CDC) include certain turtles, NHPs, bats, civets, and African rodents.

Hunting and butchering of bushmeat (for the purpose of this paper to be defined according to Oxford Dictionary as the meat of African wild animals) has been increasingly recognized as a source of disease emergence. Harvest of NHP bushmeat and exposure to NHPs in captivity have resulted in cross-species transmission of several retroviruses to humans including simian immunodeficiency virus (SIV), simian T-lymphotropic virus (STLV), and simian foamy virus (SFV) [7], [8]. While SIV and STLV adapted to humans and spread to become the global pathogens human immunodeficiency virus (HIV) and human T-lymphotropic virus (HTLV), less is known about the distribution and public health consequences of SFV infection [7]. Much of the bushmeat smuggled into the United States from Africa by air passes through Europe en route, although amount and characteristics of bushmeat reaching US borders is not well described. One study estimated that 273 tons of bushmeat was imported every year into Paris Roissy-Charles de Gaulle Airport in France on Air France carriers alone [9].

Under the authority of the Public Health Service Act, the US Department of Health and Human Service (DHHS), CDC is responsible for preventing the introduction, transmission, and spread of communicable diseases, including those from animals or animal products to humans. CDC recognizes the potential public health risk posed by illegal trade in wildlife and regulations are in place that prohibit the importation of bushmeat products derived from CDC-regulated animals. To better understand and educate the public about risks to public health from smuggled bushmeat, beginning in 2008 CDC and inter-agency and non-governmental partners initiated a cooperative effort to assess those risks. This effort includes a pilot study to screen for evidence of zoonotic pathogens in CDC-regulated wild animal products. Here we report finding sequences of simian retroviruses and herpesviruses in bushmeat confiscated at five US airports.

## Methods

### Shipment confiscation and specimen collection

This pilot study was initiated at John F. Kennedy Airport (JFK) in Queens, NY, where CDC-regulated wildlife products were seized by US Customs and Border Protection (CBP) between October 2008 to September 2010. Beginning in April 2010, additional seizures from another four airports that receive international flights (Philadelphia, Washington Dulles, George Bush Intercontinental-Houston, and Atlanta Hartsfield-Jackson International) were included in the study. Illegally imported shipments were confiscated opportunistically and thus the pilot study established only the presence and not the prevalence of zoonotic agents in the specimens.

Site of origin and destination, flight data, mail shipment or carrying passenger identification, date of arrival, and date of sample collection were recorded for each confiscation. Items were photographed and identified to genus and species if possible. Biological samples were processed for aliquoting and storage at the CDC Quarantine Laboratory at JFK Airport, and any remaining tissues were incinerated according to standard protocols. All items were sampled while wearing full personal protective equipment and sterile instruments were used to avoid cross-contamination. The freshest part of each item was located (muscle appearing red or raw, joint fluid, bone marrow, etc.) and several samples were taken from each item, placed in cryotubes, and preserved immediately in liquid nitrogen.

An additional collection of bushmeat items was seized by US Fish and Wildlife Service (USFWS) at JFK airport in 2006, and provided for this study by USFWS and the United States Geological Survey National Wildlife Health Center (NWHC). Specimens included those central to a 2006 federal case against a person caught smuggling bushmeat into New York for resale [10]. These samples had been stored at USFWS forensic laboratories at −20°C from 2006 until 2010, when they were shipped to the NWHC for processing as part of this study. All specimens were then stored at −80°C, and thawed at −20°C before processing at the NWHC. Tissue dissection was performed as described above with some minor differences; 0.5 cm^2^ samples were preserved in 1 mL Nuclisens lysis buffer (Biomerieux Inc, cat# 284135) prior to immediate storage at −80°C.

### Sample analysis and preparation

Permission was obtained from the New York Department of Agriculture and Markets to transfer the frozen specimens from JFK Airport to CDC National Center for HIV/AIDS, Viral Hepatitis, STD, and TB Prevention (NCHHSTP), and/or Columbia University's Center for Infection and Immunity (CII) for testing. When an assured gross identification of species could not be made, samples were genetically identified by phylogenetic analysis of mtDNA genes, including cytochrome *c* oxidase subunits I and II (*COX1/2*), and/or cytochrome *b* (*CytB*) [11]–[15].

Nucleic acids were extracted from 10–30 mg of tissue using mechanical disruption (Qiagen tissue lyser II or Next Advance Inc Bullet Blender), followed by proteinase K treatment until complete digestion of the tissue was achieved. Purification of subsequent homogenates was performed using the Qiagen All-Prep DNA and RNA extraction kit or DNeasy Blood and Tissue kits according to the manufacturer's instructions. Nucleic acid quality was determined using the Agilent BioAnalyser (Agilent RNA nano 6000) or ß-actin PCR as previously described [16].

### Microbial Screening

Samples were screened for multiple pathogens as described in detail elsewhere, including: leptospira and anthrax [17], herpesviruses [18], filoviruses [19], paramyxoviruses [20], coronaviruses [21], flaviviruses [22], orthopoxviruses [23] and simian retroviruses (SIV, STLV, SFV) [24]–[29]. All PCR-amplified bands approximately the expected size were confirmed by sequencing.

### Sequence Analysis

Raw sequences were analyzed and edited in Geneious Pro v5.1.7 and MEGA 5.03. Multiple sequence alignments were constructed using ClustalW and phylogenetic comparisons made using Neighbor-Joining (NJ) and maximum likelihood (ML) algorithms. ModelTest was used to select the most appropriate nucleotide substitution model. Support for branching order was evaluated using 1,000 nonparametric bootstrap support. Sequence identity was calculated using uncorrected p-distances in PAUP* and BLAST.

## Results

### Specimen condition and species composition

**Figure 1:**
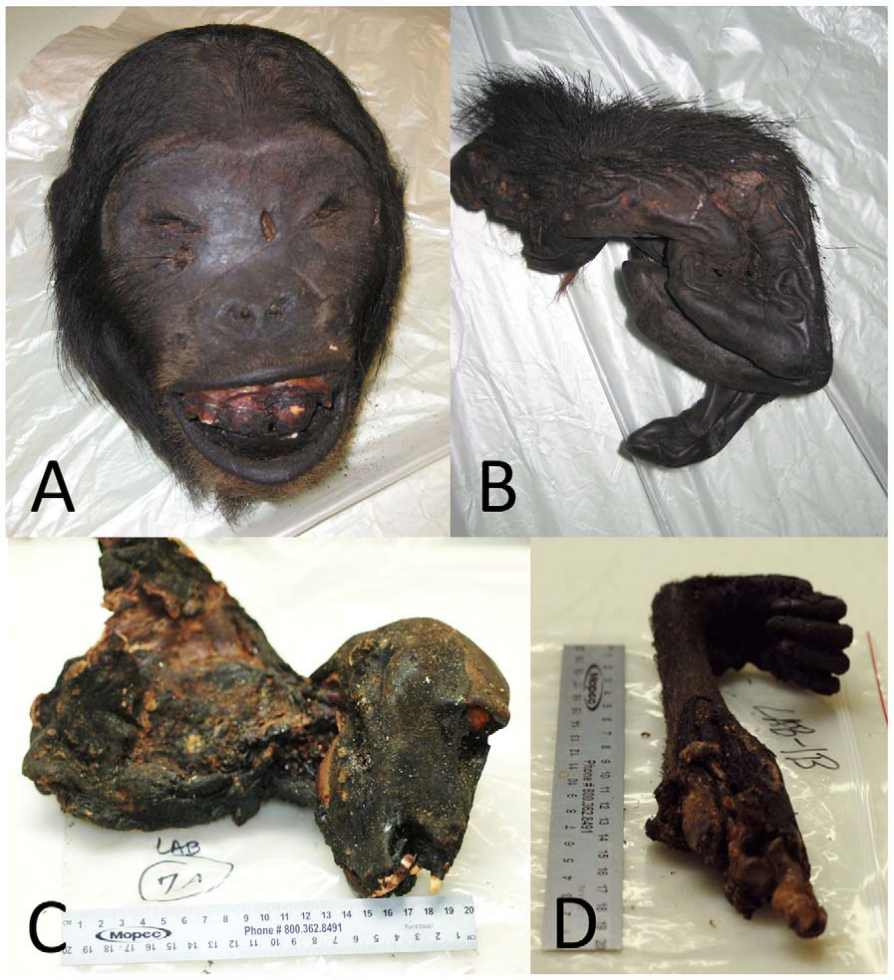
Nonhuman primate bushmeat specimens confiscated at US airports. Examples of smuggled simian bushmeat (a) skull, (b) hand, (c) skull and torso, and (d) arm. Ruler units are centimeters.

From October 2008 to September 2010, 8 postal shipments confiscated at JFK Airport were included in this study. From June 2010 to September 2010, an additional 20 passenger-carried packages confiscated at the four other international airports were sampled for this study. Additional confiscations were made but were not included in this study due to poor condition of sample (e.g., severely degraded or chemically treated). In many cases multiple separate packages were included in a single shipment or carried by a single passenger. Specimens varied in condition, including items that were fresh, raw transported in a cooler, lightly smoked, or well dried (Fig. 1A–D). Most items contained moist inner tissue. RNA quality was low with a predominance of degraded, low molecular weight fragments in the samples, while B-actin sequences were detected in the NHP specimens suggesting the presence of amplifiable DNA (data not shown). Samples from approximately 44 animals were included in this study, including 9 NHPs comprising 2 chimpanzees (*Pan troglodytes*), 2 mangabeys (*Cercocebus* spp.), and 5 guenons (*Cercopithecus* spp.; one of which was further analyzed and identified as *Cercopithecus nictitans*, white-nosed guenon) all confirmed by phylogenetic analysis; and 35 rodents comprised of 14 cane rats (*Thryonomys* sp.) confirmed by gross or phylogenetic analysis, 18 suspected cane rats (based on gross identification), and 3 rats (unknown species) confirmed by gross identification.

The USFWS specimens from 2006 included an additional 20 NHP tissues from 16 individual animals including 10 baboons (*Papio* sp.) and 6 African green monkeys (AGMs; *Chlorocebus* sp.) all confirmed by phylogenetic analysis.

### Pathogen detection

Both SFV and herpesviruses were detected in the nonhuman primate bushmeat samples. All positive NHP samples are presented in Table 1. All NHP samples were negative for SIV and STLV sequences. All rodent samples were negative for leptospira, anthrax, herpesviruses, filoviruses, paramyxoviruses, coronaviruses, flaviviruses, and orthopoxviruses.

**Table 1:** Species identification and viruses found in smuggled nonhuman primate bushmeat samples^1^.

Species^2^	Common name	Sample number^1^	Tissue	LCV	CMV	SFV	Origin of package	Destination of package
*Chlorocebus sabaeus*	green monkey	CII-040	Bone marrow	+			Guinea	Staten Island, NY
*Chlorocebus sabaeus*	green monkey	CII-051	Bone marrow	+		+	Guinea	Staten Island, NY
*Chlorocebus sabaeus*	green monkey	CII-044	Trachea	+			Guinea	Staten Island, NY
*Chlorocebus sabaeus*	green monkey	CII-144	Trachea	+			Guinea	Staten Island, NY
*Cercopithecus nictitans*	greater white-nosed monkey	BM002	Muscle		+		Nigeria	Dallas, TX
*Papio papio*	baboon	CII-013	Bone marrow	+			Guinea	Staten Island, NY
*Papio papio*	baboon	CII-028	Spinal nerve			+	Guinea	Staten Island, NY
			Muscle		+			
*Papio papio*	baboon	CII-046	Right eye			+	Guinea	Staten Island, NY
*Papio papio*	baboon	CII-163	Optic nerve		+	+	Guinea	Staten Island, NY
			Right eye	+	+			
			Trachea	+		+		
*Cercocebus atys*	sooty mangabey	BM008	Muscle	+		+	Liberia	Philadelphia, PA
*Cercocebus atys*	sooty mangabey	BM010	Muscle			+	Liberia	Philadelphia, PA
*Pan troglodytes ellioti*	Nigeria-Cameroon chimpanzee	BM013	Muscle			+	Nigeria	Queens, NY

1 Only samples testing positive are listed. All other rodent and simian samples were negative for all pathogens tested.

2 Species identification inferred with phylogenetic analysis.

### Simian Foamy Virus

**Figure 2:**
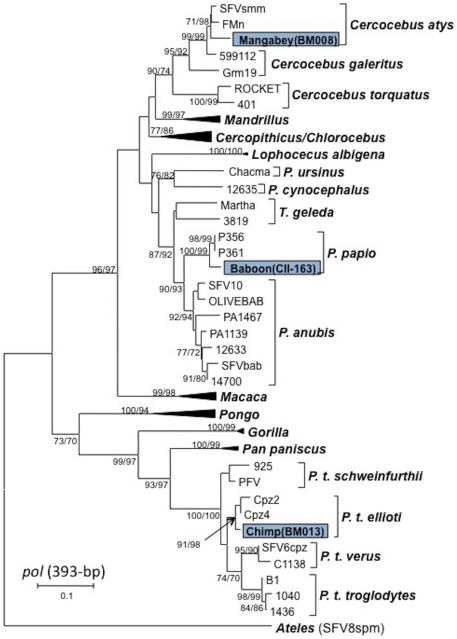
Inferred phylogenetic relationships of SFV *pol* sequences detected in bushmeat samples. Neighbor-joining (NJ) and maximum-likelihood (ML) analysis gave identical branching orders. New SFV sequences identified in this study are boxed. Clades of sequences from *Mandrillus*, *Cercopithicus*, *Chlorocebus*, *Macaca*, *Pongo*, *Gorilla*, *and Pan paniscus* are collapsed for presentation. Branch lengths are drawn to scale and only bootstrap values (NJ/ML) greater than 70% are shown.

SFV polymerase (*pol*, 465-bp) and long terminal repeat (LTR, ∼357-bp) sequences were detected at CDC in tissues from one chimpanzee (BM013) and one mangabey (BM008). SFV LTR sequences were also identified in a second mangabey (BM010). BLAST analysis of the 425-bp *pol* sequences from BM013 and BM008 showed maximum nucleotide identity to SFVs from *P. t ellioti* and mangabey (*Cercocebus atys* and *Cercocebus agilis*), respectively. Phylogenetic analysis of the two *pol* sequences with those available on GenBank confirmed that the chimpanzee SFV was highly related to SFV from *P. t. ellioti* whereas the mangabey SFV clustered tightly with SFV from sooty mangabeys (*Cercocebus atys*) (Figure 2). *P. t. ellioti* are endemic to West-Central Africa in Nigeria and Cameroon while *Cercocebus atys* are found in West Africa from Senegal to Ghana. Phylogenetic analysis was not performed on LTR sequences since only limited SFV sequences in this region are available at GenBank. BLAST analysis was similarly limited and gave the highest nucleotide identity to chimpanzee and mandrill (*M. sphinx*) SFV LTR sequences, respectively. The two LTR sequences from mangabeys (BM008 and BM010) were 94% identical to each other due to an 8-bp deletion in the LTR of BM008 and 8 nucleotide substitutions.

**Figure 3:**
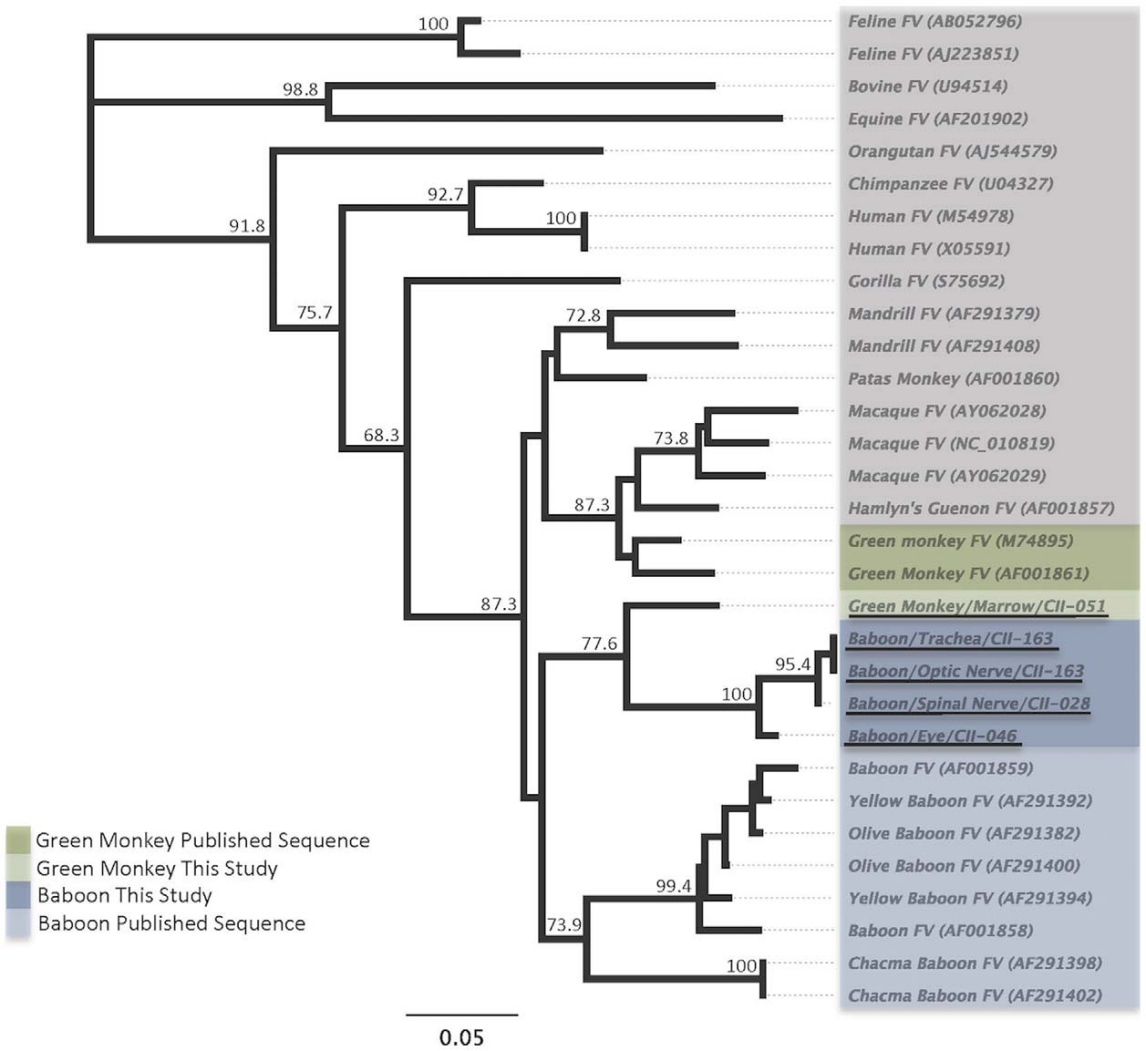
Inferred phylogenetic relationships of SFV *pol* (∼153 bp) sequences detected in USFWS bushmeat samples. Neighbor-joining (NJ) and maximum-likelihood (ML) analysis gave identical branching orders. New SFV sequences identified in this study are underlined.

In the USFWS samples SFV *pol* sequences were present in 3/10 baboons, and in 1/6 AGMs. The baboon SFVs shared >97% nucleotide identity, and had 88–90% nucleotide identity with the AGM SFV. Phylogenetic analysis of the short (156 bp) *pol* sequences shows that the three baboon SFVs clustered together, yet separately from the AGM SFV - suggesting some genetic relatedness that reflects host specificity as previously demonstrated [13] (Figure 3). However, while the short baboon SFV *pol* sequences detected in this study clustered together, they did not cluster with other published sequences from baboons (80.1–84.2% nucleotide identity). Similarly the AGM sequences did not cluster with published AGM sequences (85.8–86.5% nucleotide identity). These results may reflect poor phylogenetic signal from limited sequence data in this region.

All simian DNA samples from USFWS were also screened for larger SFV *pol* sequences (465-bp) as done at the CDC but were found in only one baboon sample (CII-163). Phylogenetic analysis of the larger *pol* sequence inferred a significant relationship to SFV from Guinea baboons (*P. papio*) (Figure 3), which correlated with the origin of the shipment (Guinea). Our inability to detect larger *pol* sequences in other SFV-positive baboon and AGM samples may be due to highly degraded nucleic acids in those specimens (confiscated in 2006) which limits detection of longer sequences.

### Herpesviruses

Two genera of herpesvirus were detected in NHP specimens, including cytomegaloviruses (CMV; betaherpesvirus) and lymphocryptoviruses (LCV; gammaherpesvirus) (Table 1). CMV sequences from baboons CII-028 and CII-163 shared >99.5% nucleotide identity indicating they are likely to be the same virus. Comparison of this virus with the CMV sequence from white-nosed guenon BM002 showed these two CMVs are 91% identical. Overall, nucleotide sequence identity within the CMVs (for sequences included here) was shown to be 68.4–100% (μ = 85.0%).

LCVs were detected in four AGMs, two baboons, and one mangabey. LCV sequences in AGMs CII-044 and CII-144 were >99% identical and likely represent the same virus. A comparison of this virus with the other LCVs detected showed 88.2–95.5% sequence identity. Sequence identity for the entire LCV group was calculated to be 81.0–100% (μ = 87.5).

**Figure 4:**
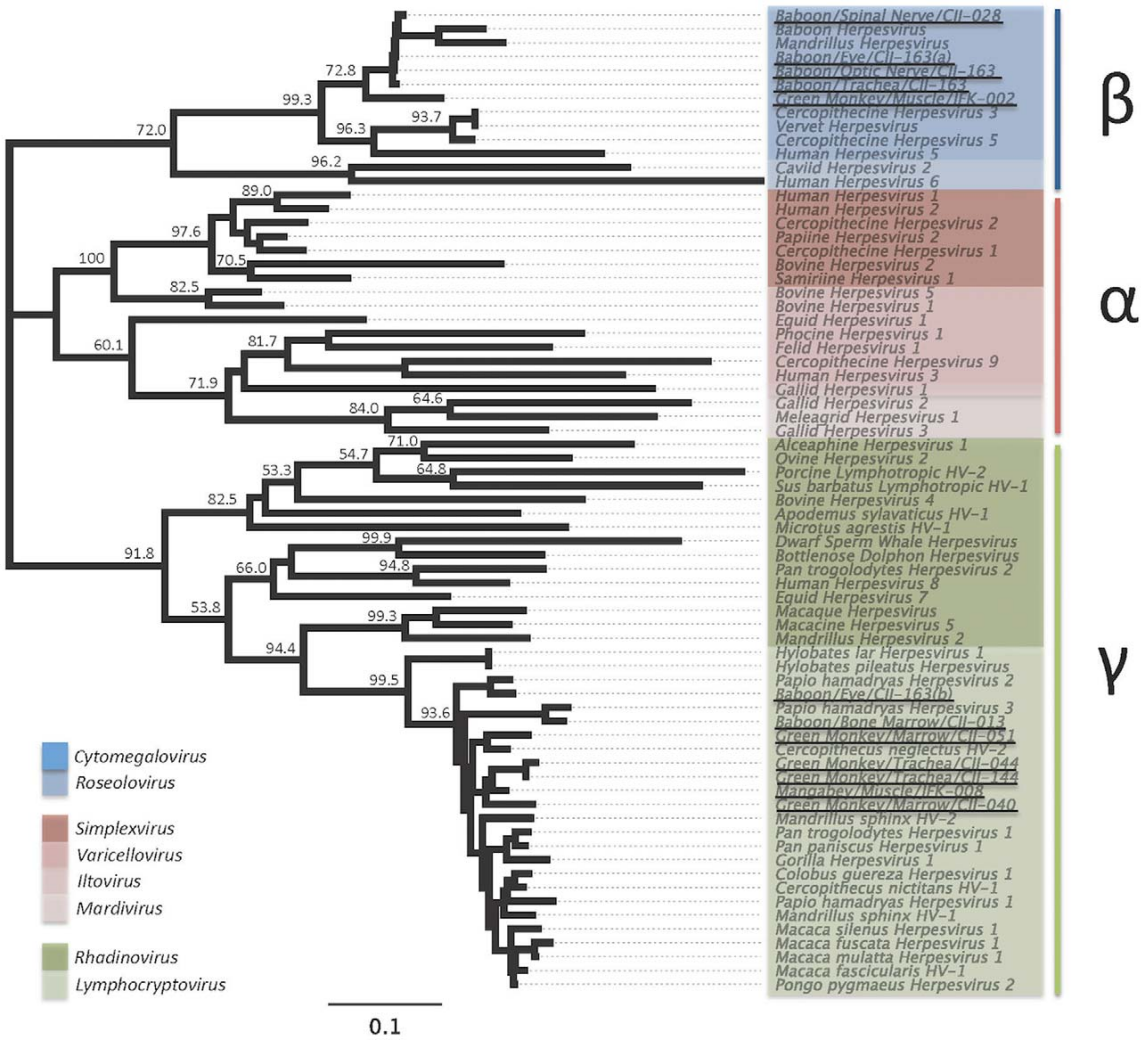
Inferred phylogenetic relationships of herpesviruses detected in siman bushmeat samples. Neighbor-joining (NJ) and maximum-likelihood (ML) analysis gave identical branching orders. Sequences identified in bushmeat products are underlined and cluster with sub-families *betaherpesvirus* (samples: CII-028, CII-163, BM-002), and *gammaherpesvirus* (samples: CII-163, CII-013, CII-051, CII-044, CII-144, CII-040, BM-008).

Phylogenetic analysis confirmed the presence and phylogenetic relatedness of CMV and LCV in these NHP specimens (Figure 4).

### Mixed infections

Multiple viruses were detected within some samples. Both LCV and SFV were detected in the bone marrow of AGM CII-051 and muscle of mangabey BM008 (Table 1). CMV, LCV, and SFV were detected in baboon CII-163 (Table 1).

### GenBank Accession numbers

New SFV, herpesvirus, and mtDNA sequences identified in the current study have been deposited at GenBank with the following accession numbers: JF810903–JF810914 and JF828317–JF828329. Sequences less than 200 bp are available upon request.

## Discussion

Our study is the first to establish surveillance for zoonotic viruses in wild animal products illegally imported into the United States in an effort to prevent the transmission of infectious agents from these shipments. The restricted number of samples included in this study were tested for a limited range of pathogens only and thus presence of additional pathogens not included in this study cannot be ruled out. We identified four SFV strains and two different herpesviruses (in some cases in the same tissues) in smuggled NHP bushmeat. Using phylogenetic analysis and gross examination, we were able to determine that bushmeat from nine NHP species and at least two rodent species were attempted to be smuggled into the United States. These results are consistent with the origin of the shipments from West Africa and included species of conservation importance (*P. papio*, *Cercocebus atys*, and *P. t. ellioti* are classified as “near threatened”, “vulnerable”, and “endangered”, respectively by the International Union for Conservation of Nature), suggesting more education efforts or harsher penalties are needed regarding the handling, consumption, and illegal transportation of products from wildlife of conservation concern. In addition, the finding of mangabey, guenon, and cane rat bushmeat in our study is consistent with that reported by Chaber *et al* who found these and bushmeat from nine other species entering Paris-Charles de Gaulle Airport [9].

Our finding of SFV DNA in smuggled NHP specimens comprising of four species (baboon, chimp, mangabey, and AGM) is significant because SFV is a known zoonotic infection of humans exposed to NHPs. However, the mode of transmission to humans is poorly understood and while most infected people reported sustaining a NHP exposure (mostly bites) others did not, suggesting a less invasive mode of infection is possible [7]. These viruses are probably not easily spread from human-to-human, although persistent infection has been documented [7]. Several SFV-positive people reported donating blood while infected and because blood banks do not screen for SFV, secondary transmission via contaminated blood donations may be possible [7]. Further research into the possibility of secondary transmission of SFV is required. The finding of SFV DNA in the bushmeat samples highlights a potential public health risk of exposure to these tissues along the hunting, transportation, and consumption continuum with multiple opportunities for primary transmissions. Unlike most retroviruses whose RNA genome is packaged in the viral particles, foamy viruses are unusual in that DNA and/or RNA can be present in the infectious virus particles. Thus, finding of only DNA does not exclude that SFV in these tissues is not infectious, especially in the more recently CDC confiscated items which contained fresher tissue compared to the USFWS items confiscated in 2006 that were partially degraded at the time of analysis in 2010. Human infection with SFV is of further concern because increases in the pathogenicity of simian retroviruses following cross-species transmission have been documented (e.g., HIV-1 and HIV-2) [30], [31]. However, the limited number of cases, short follow-up duration, and selection biases in the enrolling of healthy workers or hunters to identify cases all limit the identification of potential disease associations [7].

Although we did not find SIV or STLV in the limited number of specimens in this study, these viruses have been found in high prevalences in NHP specimens at bushmeat markets and in hunted NHPs [8], [32], [33]. HIV-1 and HIV-2 emerged as a result of several spillover events of SIV from chimpanzees and mangabeys, respectively, that were likely hunted for bushmeat in central and western Africa [30]. Serosurveillance studies have shown thirty-five different species of African NHPs harbor lentivirus infections, with a prevalence of SIV in up to 35% of free-ranging chimpanzees, and 30–60% of free-ranging sooty mangabeys and green monkeys [30], [31], [33], [34].

To date, four groups of HTLV viruses found in humans are believed to have originated from corresponding STLV strains in NHP species (including mangabeys, baboons, and chimpanzees) via multiple transmission events [35]. HTLV-1, closely related to STLV-1 group viruses, infects 15 to 20 million people worldwide and is spread from person to person via bodily fluids [35]. These viruses are capable of causing leukemia, lymphoma and neurologic disease in humans [35]. Discoveries of HTLV-3 and HTLV-4, and a novel STLV-1 strain were recently made in NHP hunters in Cameroon [7], and 89% of hunted bushmeat in Cameroon has been shown to be infected with STLV strains [8], [32]. Although imported wildlife products are often not in a freshly-killed state, many are not smoked or processed in any manner, thus screening of larger sample collections of smuggled bushmeat may reveal evidence of these viruses.

Like retroviruses, herpesviruses can cause long-term latent infections in their host. Most herpesviruses are host-specific, yet particular strains are capable of causing severe disease in the non-host, examples of which include agents of malignant catarrhal fever and Herpes B virus [36], [37]. CMVs are in the betaherpesvirus subfamily. Human CMV is typically asymptomatic in humans, with the exception of immunocompromised persons. Similarly, many NHPs are asymptomatic hosts of CMV that do not typically infect other species, including humans. However, baboon CMV (bCMV), like that identified in our study, has been shown to replicate in human tissues in vitro as well as infect and replicate in humans following a bCMV-positive liver xenotransplant [38].

Lymphocryptoviruses (LCV) are in the gammaherpesvirus subfamily, and include human LCV, and Epstein-Barr virus (EBV), the agent of infectious mononucleosis. Nearly 90% of adults in the United States have antibodies indicating exposure at some point to EBV. LCVs are typically asymptomatic in their host, with the exception of immunocompromised individuals who may develop B-cell tumors. Although much less efficient, baboon LCV can infect human B cells in immunocompromised persons or in persons co-infected with EBV and replicate in EBV-immortalized B cells with the theoretical potential for viral recombination [39]. However, it is unknown if the novel herpesviruses found in bushmeat specimens in our study can easily infect humans handling these tissues. Systematic studies examining herpesvirus transmission risks associated with handling or consumption of infected animal tissues have not been reported. In addition, virus isolation was not performed in our study to determine the infectiousness of the specimens at the time of confiscation.

In summary, our study establishes initial surveillance methodology to detect and identify zoonotic pathogens and species of origin of wildlife products entering the United States. While we were successful in demonstrating the presence of SFV and herpesviruses in bushmeat specimens, our pilot study was limited by the range, number, and variable condition of products available to us and was not intended to be a comprehensive review of presence or to measure prevalence of all pathogens imported in wildlife products. Because our study only included a small number of CDC-regulated species and excluded products of ungulate, carnivore, reptile, avian and other origin, as well as any live animal imports, all of which may carry zoonotic pathogens or diseases that threaten domestic livestock or native wildlife, in addition to the fact that virus isolation was not performed in our study to determine the infectiousness of the specimens at the time of confiscation, there is a large component of zoonotic disease risk assessment not included in this study. A further understanding of pathogen movements through the trade will only be recognized through broader surveillance efforts and pathogen identification and discovery techniques in wildlife and wildlife products arriving at US ports of entry so that appropriate measures can be taken to further mitigate potential risks.
